# The association between plasma furin and cardiovascular events after acute myocardial infarction

**DOI:** 10.1186/s12872-021-02029-y

**Published:** 2021-09-27

**Authors:** Zhi-Wei Liu, Qiang Ma, Jie Liu, Jing-Wei Li, Yun-Dai Chen

**Affiliations:** 1 Department of Cardiology, People’s Liberation Army General Hospital, No. 28 Fuxing Road, Wukesong, Haidian District, Beijing, 100853 China; 2 The Second Afliated Hospital and Yuying Children’s Hospital Wenzhou Medical University, Wenzhou, 325027 China; 3 Department of Cardiology, Xinqiao Hospital, Third Military Medical University, Chongqing, China

**Keywords:** Furin, Acute myocardial infarction, Major adverse cardiac events

## Abstract

**Background:**

Furin is the key enzyme involved in the cleavage of pro-BNP and plays a critical role in the cardiovascular system through its involvement in lipid metabolism, blood pressure regulation and the formation of atheromatous plaques. NT-proBNP and recently, corin, also a key enzyme in the cleavage of pro-BNP, have been accepted as predictors of prognosis after acute myocardial infarction (AMI). This cohort study was conducted to investigate the relationship between plasma furin and the prognostic outcomes of AMI patients.

**Methods:**

In total, 1100 AMI patients were enrolled in the study and their plasma furin concentrations were measured. The primary endpoint was major adverse cardiac events (MACE), a composite of cardiovascular (CV) death, non-fatal myocardial infarction (MI) and non-fatal stroke. The associations between plasma furin concentration and AMI outcomes were explored using Kaplan–Meier curves and multivariate Cox regression analysis.

**Results:**

The results showed a slight increase in mean cTNT in patients with higher furin concentrations (*P* = 0.016). Over a median follow-up of 31 months, multivariate Cox regression analysis indicated that plasma furin was not significantly associated with MACE (HR 1.01; 95% CI 0.93–1.06; *P* = 0.807) after adjustment for potential conventional risk factors. However, plasma furin was associated with non-fatal MI (HR 1.09; 95% CI 1.01–1.17; *P* = 0.022) in the fully adjusted model. Subgroup analyses indicated no relationship between plasma furin and MACE in different subgroups.

**Conclusions:**

This study found no association between plasma furin and risk of MACE. Thus, plasma furin may not be a useful predictor of poor prognosis after AMI. However, higher levels of plasma furin may be associated with a higher risk of recurrent non-fatal MI.

**Supplementary Information:**

The online version contains supplementary material available at 10.1186/s12872-021-02029-y.

## Introduction

Cardiovascular diseases (CVDs) remain a major cause of premature death and chronic disability across all regions in the world [1]. Acute myocardial infarction (AMI) is a severe CVD. The currently available scoring systems, such as the Global Registry of Acute Coronary Events (GRACE) and the Thrombolysis In Myocardial Infarction (TIMI), have been established to assist clinicians with the selection of treatment strategies for AMI patients at an early stage [2, 3]. Troponin I and NT-proBNP have been accepted as predictors of prognosis after AMI [4, 5]. However, new biomarkers may be helpful in precisely predicting poor prognosis or may contribute to a better understanding of the pathological process of AMI patients.

A recent study reported that corin was an independent predictor of prognosis in patients with AMI [6]. Furin, another core enzyme that cleaves proBNP into active BNP fragments and corin [7], may be associated with poor prognosis after AMI.

Furin is a mammalian subtilisin/kex2p-like endoprotease involved in the processing of various precursor proteins [8]. Studies have shown that furin plays an important role in the cardiovascular system through regulation of lipid and cholesterol metabolism, blood pressure (BP) and the formation of atherosclerotic lesions [9]. Michael T et al. found that circulating furin cleaved proprotein convertase subtilisin/kexin type 9 (PCSK9); PCSK9 regulates LDL receptors and serum atheromatous plaques [10, 11]. Furin is also involved in BP regulation by shedding endogenous (pro)renin receptors [12], promoting the migration and proliferation of vascular smooth muscle cells [13] and activating the epithelial Na + channel [14]. Moreover, Gopala et al. [15] observed that inhibition of furin in the atherosclerotic segment of mice decreased vascular remodelling and atherosclerosis. Furin is a better predictor of cardiovascular (CV) outcomes than BNP and corin in type 2 diabetes patients [16]. Further, limited research has suggested that furin may be associated with CV events after MI [17]. Therefore, this study was performed to evaluate the prognostic utility of plasma furin in AMI patients.

## Methods

### Study population

A total of 1100 AMI patients consecutively admitted to the People’s Liberation Army General Hospital (PLAGH) between January 2013 and September 2017 were included in this study. All participants provided written informed consent. This study was approved by the institutional review board of the PLAGH and was performed in accordance with the Declaration of Helsinki. AMI was diagnosed if the patient had a cardiac troponin I level exceeding the 99th percentile of a normal reference population with at least one of the following: chest pain lasting > 20 min, diagnostic serial electrocardiographic changes consisting of new pathologic Q waves, or ST-segment and T-wave changes [18].

### Biochemical measurements

Researchers who were blinded to the patients' characteristics and outcomes conducted biochemical measurements. Blood samples were collected from the AMI patients on the first morning after admission. Plasma was obtained by centrifugation for 10 min at 3,000 rpm and then stored at -80 °C until further analysis. Plasma furin concentrations were determined in EDTA-treated plasma samples using a commercially available kit (Catalog # EHFURIN, ThermoFisher, USA), according to the manufacturer’s instructions.

### Outcome events and follow-up

The clinical, demographic and biochemical data of the patients were obtained from the hospital files and computer records. The primary endpoint for this study was major adverse cardiac events (MACE), a composite of CV death, non-fatal myocardial infarction (MI) or non-fatal stroke. Other endpoints of interest included hospitalisation for heart failure (HF), non-CV death and all-cause death. Hospitalisation for HF was defined as a hospital readmission primarily due to HF. Recurrent MI was diagnosed in accordance with established criteria, as described [18]. The endpoints were obtained by reviewing the clinical records of the re-admitted patients or by contacting each patient individually.

### Statistical analyses

Continuous variables were compared using the Kruskal–Wallis test. Categorical variables were expressed as counts (percentages) and compared using the Chi-square test. The correlation analysis was performed using the Spearman method. The associations between plasma furin concentration and AMI outcomes were explored using the Kaplan–Meier method with stratification by furin tertile. The results were also evaluated with Cox proportional hazard regression models. The adjusted covariates included in the multivariate models have been previously shown to be associated with MACE. Model 1 was adjusted for age and sex. Model 2, the fully adjusted model, was also adjusted for: eGFR, BMI, smoking, history of diabetes, hypertension and MI, and STEMI/non-STEMI. Subgroup analyses were also undertaken to determine whether furin was associated with MACE in different age, gender, BMI, smoking status, diabetes, hypertension and STEMI/NSTEMI subgroups. Multiple linear regression analysis was performed to identify variables independently associated with furin among the entire study sample. All statistical tests were two-tailed and a *P* value less than 0.05 was considered statistically significant. All analyses were performed with SAS version 9.4.

## Results

### Baseline data

The mean age of the 1,100 study participants was 61 ± 13 years; 77% were male. The distribution of plasma furin was left-skewed (Additional file 2: Figure S1). The median plasma furin level was 156.6 (interquartile range, 102.4–228.8) pg/ml. There was no significant difference between male and female patients (158.5 [103.4–226.9] pg/ml for males versus 145.9 [93.1–233.6] pg/ml for females; *P* = 0.360), between diabetic and non-diabetic patients [160.9 (104.2–231.0] pg/ml for diabetics versus 155.1 [101.6–224.9] pg/ml for non-diabetics; *P* = 0.535), between hypertensive and non-hypertensive patients (154.0 [101.7–222.6] pg/ml for hypertensive versus 160.8 [103.0–232.1] pg/ml for non-hypertensive; *P* = 0.233) and between STEMI and non-STEMI patients (160.7 [105.3–231.4] pg/ml for STEMI versus 147.2 [94.9–220.1] pg/ml for NSTEMI; *P* = 0.079).

### Associations between plasma furin levels and clinical parameters

The baseline characteristics of the sample are listed in Table 1. AMI patients were divided into three groups according to their plasma furin tertile (≤ 117.5 pg/ml, 117.5–200 pg/ml, ≥ 200 pg/ml). There was a slight increase in the mean cTNT in patients with higher furin levels (*P* = 0.016). There was no significant increase in NT-proBNP as plasma furin increased (Table 1).

**Table 1:** Baseline variables according to the plasma furin tertile of AMI patients

		Plasma furin (pg/mL)			*P* value
	Overall	≤ 117.5	117.5–200	≥ 200	
Patients, n	1100	356	374	370	
Anterior MI, n (%)	313 (29.6%)	100 (29.1%)	116 (32.4%)	97 (27.2%)	0.300
STEMI, n (%)	747 (69.6%)	231 (66.8%)	256 (70.7%)	260 (72.2%)	0.265
Age, year	61.0 (13.4)	60.8 (13.8)	61.6 (13.0)	60.7 (13.4)	0.489
Male, n (%)	817 (77.0%)	262 (75.9%)	275 (76.6%)	280 (78.4%)	0.718
Current smoker, n (%)	370 (41.9%)	127 (44.1%)	116 (38.5%)	127 (34.1%)	0.345
*Medical history, n (%)*					
Diabetes mellitus	394 (37.1%)	122 (35.7%)	140 (38.8%)	132 (36.9%)	0.690
Hypertension	456 (43.2%)	150 (44.0%)	159 (44.5%)	147 (41.2%)	0.624
MI	45 (4.1%)	14 (3.9%)	13 (3.5%)	18 (4.9%)	0.623
CKD	20 (1.9%)	8 (2.4%)	7 (2.0%)	5 (1.4%)	0.654
AF	10 (0.9%)	4 (1.1%)	4 (1.1%)	2 (0.5%)	0.648
LIPID	121 (11.0%)	40 (11.2%)	38 (10.2%)	43 (11.6%)	0.828
HF	5 (0.5%)	4 (1.1%)	0 (0.0%)	1 (0.3%)	0.090
*Clinical assessment*					
BMI (kg/m^2^)	25.3 (3.6)	25.4 (3.8)	25.5 (3.7)	24.9 (3.3)	0.079
HBA1C (%)	6.7 (1.6)	6.7 (1.6)	6.8 (1.6)	6.6 (1.6)	0.465
Glucose (mmol/L)	8.5 (3.9)	8.3 (3.5)	8.4 (4.0)	8.7 (4.3)	0.944
Cr (µmol/L)	78.5 (68.0, 94.7)	79.1 (67.6, 95.6)	78.1 (67.6, 93.9)	78.0 (68.7, 93.8)	0.912
LVEF (%)	50.5 (9.0)	51.0 (9.0)	50.1 (9.3)	50.5 (8.7)	0.509
cTNT (pg/mL)	1.8 (0.5, 4.9)	1.4 (0.4, 3.9)	1.7 (0.5, 4.6)	2.1 (0.6, 6.2)	0.016
NT-proBNP (pg/mL)	1566(668, 3929)	1427 (610, 3490)	1587 (712, 4098)	1645 (709, 4049)	0.361
HR	77.6 (14.9)	76.5 (13.5)	77.3 (14.2)	78.8 (16.7)	0.591
CHOL (mmol/L)	4.3 (1.1)	4.2 (1.1)	4.4 (1.1)	4.2 (1.1)	0.601
TRIG (mmol/L)	1.3 (0.9, 1.8)	1.3 (0.9, 1.8)	1.3 (1.0, 1.9)	1.3 (0.9, 1.8)	0.453
LDL (mmol/L)	2.7 (0.9)	2.6 (0.9)	2.7 (1.0)	2.7 (0.9)	0.588
HDL (mmol/L)	1.1 (0.3)	1.1 (0.3)	1.1 (0.3)	1.1 (0.3)	0.771
AST (U/L)	45.6 (24.5, 108.3)	42.1 (24.3, 91.1)	43.9 (24.4, 100.3)	52.0 (26.6, 132.7)	0.112
ALT (U/L)	30.8 (19.6, 52.5)	31.6 (19.4, 51.7)	30.7 (19.7, 51.1)	31.1 (19.6, 55.1)	0.898
GGT (U/L)	29.1 (19.3, 47.9)	28.4 (19.3, 48.5)	28.9 (19.3, 47.1)	29.7 (19.4, 51.0)	0.772
PT (s)	14.1 (2.0)	14.0 (2.1)	14.0 (1.3)	14.3 (2.3)	0.114
APTT (s)	39.4 (35.3, 46.8)	38.8 (35.3, 45.6)	39.3 (35.1, 45.9)	39.9 (35.4, 51.5)	0.304
DDIMER (ng/L)	0.4 (0.3, 0.8)	0.4 (0.3, 0.8)	0.4 (0.3, 0.8)	0.4 (0.3, 0.9)	0.653
*Medications, n (%)*					
Aspirin	1021 (96.3%)	334 (97.1%)	347 (96.7%)	340 (95.2%)	0.392
ACEI/ARB	434 (40.9%)	146 (42.4%)	158 (44.0%)	130 (36.4%)	0.093
Statin	1033 (97.5%)	338 (98.3%)	351 (97.8%)	344 (96.4%)	0.251
Diuretic	572 (52.0%)	186 (52.2%)	193 (51.6%)	193 (52.2%)	0.996
Cablocker	121 (11.0%)	45 (12.6%)	41 (11.0%)	35 (9.5%)	0.394
Betablocker	528 (48.0%)	187 (52.5%)	178 (47.6%)	163 (44.1%)	0.070
GLP1	22 (2.0%)	6 (1.7%)	8 (2.1%)	8 (2.2%)	0.875
Insulin	531 (48.3%)	158 (44.4%)	182 (48.7%)	191 (51.6%)	0.146
DPP4	49 (4.5%)	15 (4.2%)	16 (4.3%)	18 (4.9%)	0.895

Data are presented as mean (SD), median (interquartile range) or number (percentages). *ACEI* angiotensin converting enzyme inhibitor, *ARB* angiotensin receptor blocker, *BMI* body mass index, *cTNT* cardiac troponin T, *eGFR* estimated glomerular filtration rate, *LVEF* left ventricular ejection fraction, *MI* myocardial infarction, *STEMI* ST-elevation myocardial infarction

Spearman correlation analysis showed that log-transferred furin was not significantly correlated with age, blood glucose, HbA1c, left ventricular ejection fraction, log eGFR, log cTNT, log CKMB or log NT–proBNP (Additional file 1: Table S1).

### Kaplan–Meier analysis

**Fig. 1:**
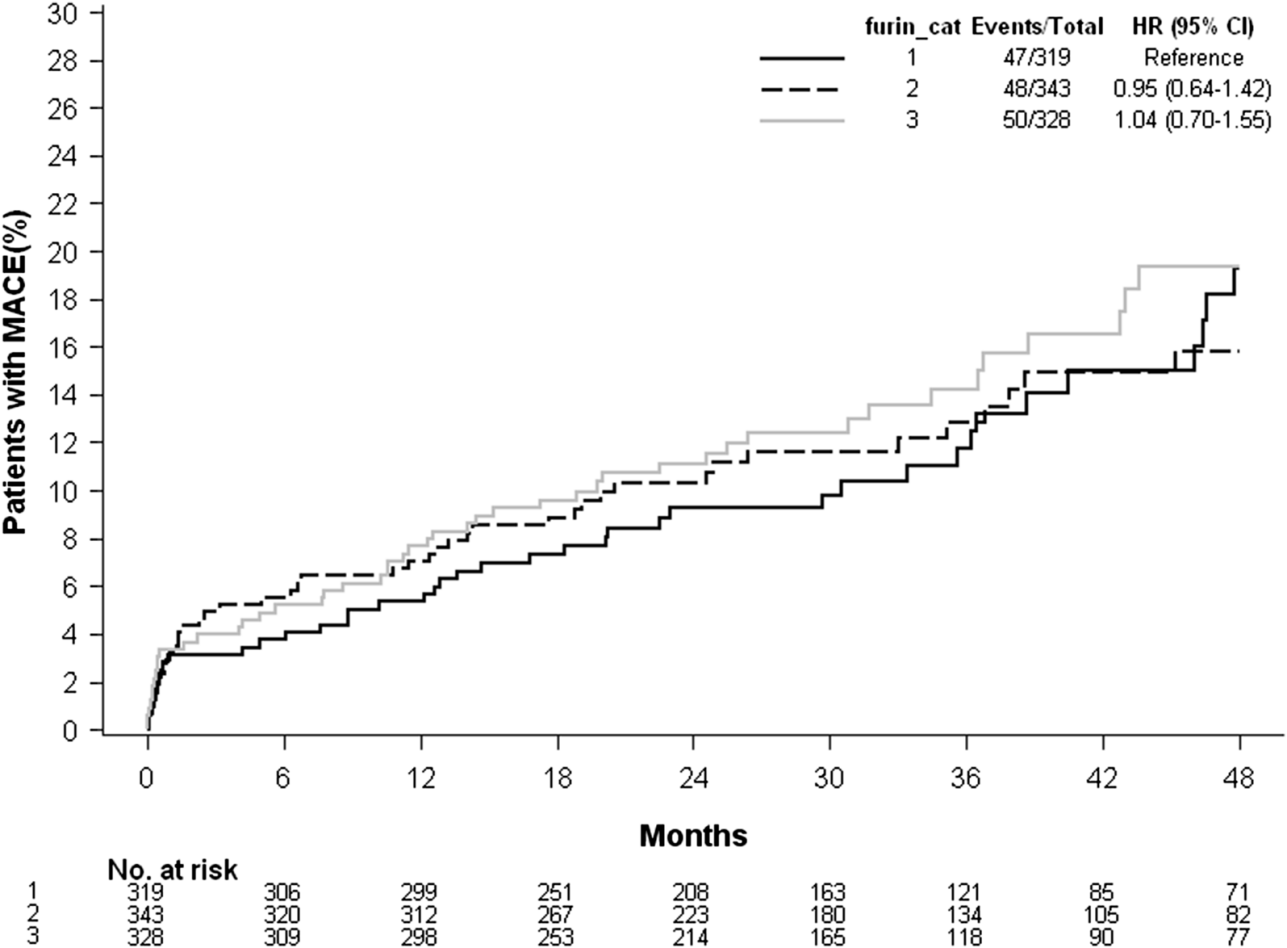
Kaplan–Meier analysis of MACE rates in AMI patients according to different furin categories

Over a median follow-up of 31 months, 133 cases of cardiovascular death, 37 cases of non-cardiovascular death, 26 cases of recurrent non-fatal MI, 22 cases of non-fatal stroke and 27 cases of hospitalisation for HF occurred in the sample. Kaplan–Meier survival analysis suggested that furin was not associated with the composite CV outcome (Fig. 1).

### COX regression analysis of endpoints

Cox regression analysis indicated that increasing plasma furin level was not associated with an increased risk of MACE (HR 1.01; 95% CI 0.93–1.06; *P* = 0.807). In addition, each endpoint of CV death, non-fatal MI, non-fatal stroke, non-CV death, all death or hospitalisation for HF was investigated. The results showed that plasma furin was not associated with any of these endpoints except for possibly a higher risk of recurrent non-fatal MI (HR 1.09; 95% CI 1.01–1.17; *P* = 0.022) (Table 2).

**Table 2:** Effect of every 50-unit increase in furin on cardiovascular outcomes

		Unadjusted		Model 1		Model 2	
	Event/no	HR (95% CI)	*P* value	HR (95% CI)	*P* value	HR (95% CI)	*P* value
*MACE*							
Low (≤ 117.5)	60/356	1.12 (0.80, 1.56)	0.519	1.18 (0.84, 1.66)	0.338	1.41 (0.91, 2.17)	0.125
Medium (117.5–200)	57/374	Ref		Ref		Ref	
High (≥ 200)	64/370	1.04 (0.74, 1.46)	0.824	1.08 (0.77, 1.53)	0.655	1.20 (0.76, 1.90)	0.433
50-pg/mL increase	181/1100	1.01 (1.00, 1.03)	0.084	1.02 (1.00, 1.03)	0.030	1.01 (0.96, 1.06)	0.807
*CV death*							
Low (≤ 117.5)	46/356	1.17 (0.77, 1.78)	0.465	1.21 (0.79, 1.86)	0.376	1.29 (0.75, 2.22)	0.350
Medium (117.5–200)	42/374	Ref		Ref		Ref	
High (≥ 200)	45/325	1.10 (0.72, 1.67)	0.661	1.20 (0.78, 1.84)	0.410	1.02 (0.57, 1.83)	0.939
50-pg/mL increase	133/1100	1.01 (1.00, 1.03)	0.078	1.02 (1.00, 1.04)	0.013	0.99 (0.92, 1.06)	0.709
*Non-fatal MI*							
Low (≤ 117.5)	7/356	1.16 (0.41–3.30)	0.786	1.20 (0.42, 3.45)	0.734	1.68 (0.36, 7.90)	0.509
Medium (117.5–200)	7/374	Ref		Ref		Ref	
High (≥ 200)	12/370	1.67 (0.66, 4.25)	0.280	1.73 (0.68, 4.45)	0.253	5.12 (1.24, 21.2)	0.024
50-pg/mL increase	26/1100	1.02 (0.98, 1.07)	0.394	1.02 (0.98, 1.07)	0.316	1.09 (1.01, 1.17)	0.022
*Non-fatal stroke*							
Low (≤ 117.5)	7/356	1.04 (0.38, 2.86)	0.945	0.99 (0.35, 2.74)	0.976	1.34 (0.41, 4.40)	0.625
Medium (117.5–200)	8/374	Ref		Ref		Ref	
High (≥ 200)	7/370	0.88 (0.32, 2.43)	0.806	0.83 (0.29, 2.41)	0.731	0.62 (0.15, 2.65)	0.521
50-pg unit increase	22/1100	0.92 (0.76, 1.11)	0.389	0.91 (0.74, 1.12)	0.358	0.85 (0.64, 1.14)	0.277
*Hospitalised HF*							
Low (≤ 117.5)	10/356	1.05 (0.44, 2.53)	0.908	1.08 (0.45, 2.60)	0.860	1.85 (0.53, 6.39)	0.333
Medium (117.5–200)	10/374	Ref		Ref		Ref	
High (≥ 200)	7/370	0.71 (0.27, 1.85)	0.479	0.74 (0.28, 1.94)	0.540	1.71 (0.48,6.10)	0.405
50-pg/mL increase	27/1100	0.99 (0.91, 1.09)	0.862	0.99 (0.90, 1.09)	0.876	1.03 (0.94,1.14)	0.490
*Non-CV death*							
Low (≤ 117.5)	16/356	1.68 (0.76, 3.71)	0.197	1.88 (0.85, 4.15)	0.121	1.70 (0.60, 4.79)	0.316
Medium (117.5–200)	10/374	Ref		Ref		Ref	
High (≥ 200)	11/370	1.11 (0.47, 2.61)	0.817	1.15 (0.49, 2.71)	0.097	0.58 (0.14, 2.39)	0.449
50-pg/mL increase	37/1100	1.01 (0.99, 1.04)	0.305	1.02 (1.00, 1.05)	0.093	0.94 (0.75, 1.17)	0.561
*All death*							
Low (≤ 117.5)	62/356	1.28 (0.89, 1.85)	0.191	1.36 (0.93, 1.98)	0.112	1.37 (0.85, 2.22)	0.195
Medium (117.5–200)	52/374	Ref		Ref		Ref	
High (≥ 200)	56/370	1.11 (0.76, 1.62)	0.600	1.21 (0.83, 1.78)	0.327	0.94 (0.55, 1.59)	0.804
50-pg/mL increase	170/1100	1.02 (1.00,1.03)	0.025	1.02 (1.01, 1.04)	0.002	0.98 (0.92, 1.05)	0.548

Model 1 adjusted for age and sex

Model 2 adjusted for model 1 plus eGFR, BMI, smoking, history of diabetes, hypertension or MI, and STEMI/non-STEMI

### Subgroup analysis

**Fig. 2:**
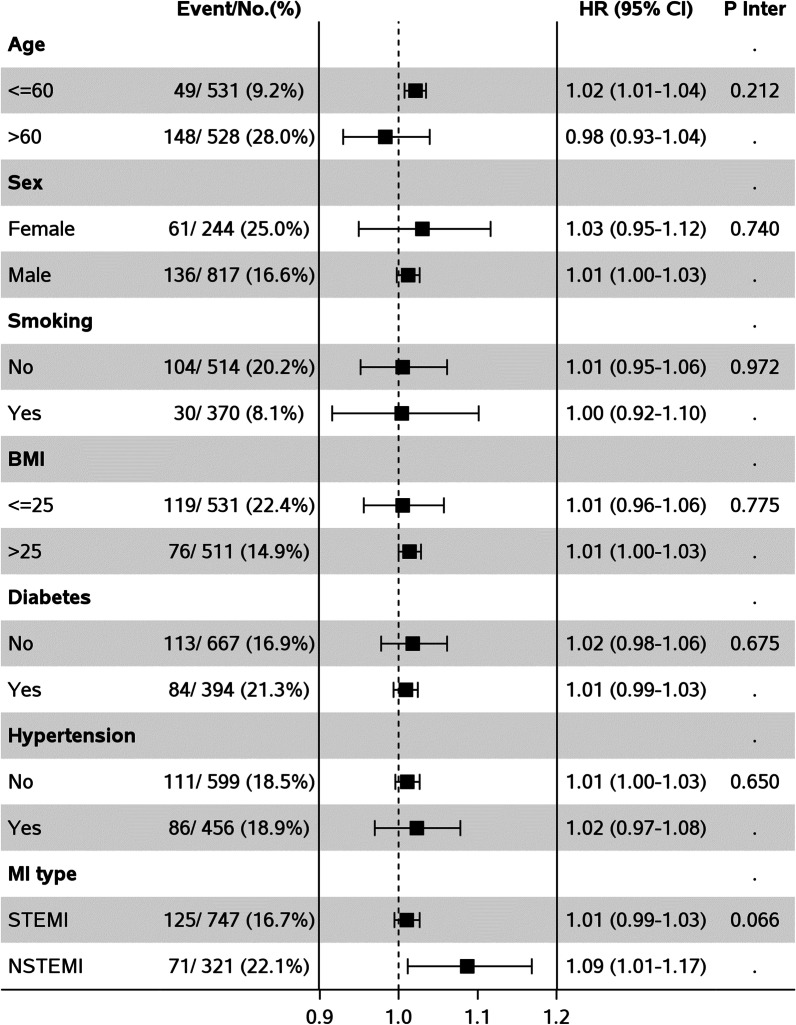
Association between furin and cardiovascular outcomes among subgroups of patients

Subgroup analysis showed that the association between furin and MACE did not differ according to age, gender, BMI, history of smoking, diabetes, hypertension and type of MI (STEMI/NSTEMI) (Fig. 2).

Finally, univariable cox regression analysis was performed to identify variables that may be independently associated with MACE in the sample (Table 3). The results showed that NT–proBNP (*P* < 0.001), age (*P* < 0.001), creatinine (*P* < 0.001), cTnT (*P* = 0.001), blood glucose (*P* = 0.001), diabetes history (*P* = 0.010), CKD history (*P* = 0.023) and STEMI (*P* = 0.039) were all positively associated with MACE. In contrast, LVEF (*P* < 0.001), usage of aspirin (*P* < 0.001), ACEI/ARB (*P* < 0.001) and male sex (*P* = 0.001) were negatively associated with MACE.

**Table 3:** Univariable predictors of MACE after MI in the whole sample

Predictor	Chi-Square	HR (95%CI)	*P*
NT-proBNP (1000 pg/ml greater)	240.4268	1.09 (1.08, 1.11)	< 0.001
Age (year older)	120.0124	1.07 (1.05, 1.08)	< 0.001
LVEF (1% greater)	94.6846	0.93 (0.91, 0.94)	< 0.001
Creatinine(10-unit increase)	21.6745	1.02 (1.01–1.03)	< 0.001
Aspirin (yes/no)	14.0069	0.38 (0.23, 0.63)	< 0.001
ACEI/ARB (yes/no)	12.1239	0.57 (0.42, 0.78)	< 0.001
cTNT (1 μg/L greater)	11.4552	1.03 (1.01, 1.04)	0.001
Glucose (1 mg/dL greater)	11.4426	1.05 (1.02, 1.07)	0.001
Male	10.3531	0.61 (0.45, 0.82)	0.001
Diabetes (yes/no)	6.6461	1.45 (1.09, 1.93)	0.010
CKD (yes/no)	5.1332	2.40 (1.13, 5.10)	0.023
STEMI	4.2433	1.36 (1.01, 1.82)	0.039
Hypertension (yes/no)	2.6197	1.27 (0.95, 1.69)	0.106
Furin (50 pg/mL greater)	2.1521	1.01 (1.00, 1.03)	0.142
Hba1c (1 unit greater)	1.7690	1.07 (0.97, 1.19)	0.184
Statin (yes/no)	0.0710	0.90 (0.40, 2.02)	0.790

## Discussion

This study of 1,100 consecutive AMI patients demonstrated that plasma furin was not associated with MACE events, but may be associated with a higher risk of non-fatal MI.

Furin is an enzyme that converts various inactive protein precursors into their active forms. In lipid metabolism, furin-cleaved PCSK9 increases the LDL receptor, leading to a decrease in LDL-C [11, 19]. On the other hand, ANGPTL 3 and 4, which are also cleaved by furin, can mediate endothelial lipase and lipoprotein lipase inactivation [9, 20]. The renin receptor, which is activated by furin, binds to renin or prorenin and consequently increases BP [21]. The epithelial Na + channel (ENaC), another substrate of furin, is associated with increased BP [14, 22]. On the other hand, transforming growth factor is also activated by furin but it may contribute to lowering BP [23, 24]. BNP activated by furin is also associated with low BP through its diuretic and vasodilatory actions. Together, these findings indicate that the underlying mechanisms of plasma furin in the cardiovascular system may be complex and bi-directional.

Clinical studies investigating the role of furin in the cardiovascular system have also produced inconsistent findings. Li et al. suggested that the furin gene may be a candidate gene involved in human hypertension as the G allele of 1970C > G is a modest risk factor for hypertension [25]. However, another human genome-wide association study found that the AA genotype of rs4702 in the furin gene, which leads to less furin protein expression, was associated with both elevated SBP and DBP [26]. A study comprising 4678 healthy European adults found that higher baseline plasma furin was significantly associated with higher BMI, blood glucose and BP [27]. However, another study comprising 2312 healthy Chinese adults found inverse associations between furin and both blood glucose and BP [28]. In the current study, there were no significant relationships between plasma furin and BMI, BP or blood glucose. These findings suggest that furin may play a complicated role in the cardiovascular system in certain conditions. A recent paper reported that plasma furin was positively associated with MACE after MI [17]; however, the methods for blood sample collection were not described in detail and thus, this study cannot be directly compared with the current study. Moreover, in this previous study, the primary endpoint, MACE, was defined as all-cause mortality, hospitalisation for HF and recurrent MI, while in the current study it included CV death, non-fatal MI and non-fatal stroke [28]. These differences could have led to the differing results of the two studies. Mechanistic studies of furin in CVDs should further clarify its activity and regulatory factors.

NT-proBNP provides prognostic value for MACE in patients with AMI [4], and this was verified in the current study. Glycosylation and an increase in furin activity are two major post-translational modifications that reduce proBNP [29]; these synergistically lead to increased circulating BNP and NT-proBNP. It should be mentioned that neither the concentrations of corin/furin nor the corin activity increase during this process [29]. It is possible that an increase in furin activity but not plasma furin concentration is pivotal in the increase in circulating BNP in AMI.

The current study found a potential association between plasma furin concentration and recurrent non-fatal MI after adjustment for conventional risk factors. The results of previous studies investigating the role of furin in atherosclerosis may explain this potential association. Furin mRNA was found to be increased after MI in a rat model and the expression of furin was negatively correlated with the LVEF [30, 31]. In addition, over-expression of furin was found in human atherosclerotic plaques and inhibition of furin was found to decrease vascular remodelling and atherosclerosis in mouse models, suggesting that furin may play an important role in plaque progression [32]. Furin activates many pro-inflammatory cytokines, such as TNF-α and IFN-γ, which leads to the progression of atherosclerosis [33, 34]. Furthermore, furin levels are associated with higher circulating MCP1 levels and greater carotid intima-media thickness [35]. In the current study, a slight increase in cTNT was found in patients with higher furin levels. Higher peak concentrations of cTnT reflect a larger infarct area [5]. It is possible that higher levels of furin may indicate progression of atherosclerosis and more severe or vulnerable plaque lesions, resulting in a higher risk of recurrent non-fatal MI.

The current results indicated that plasma furin concentration was not associated with the risk of MACE but may be associated with non-fatal MI. Studies with larger sample sizes are needed to verify these results and detailed basic studies are required to further explore these findings. There are several limitations of this study that should be noted. First, this cohort study was conducted at a single centre among the Chinese population; the generalisability of these findings to other populations with different genetic backgrounds and health profiles should be performed with caution. Second, blood samples were collected on the first morning after admission. There were no samples available at other time points after MI. The dynamic changes in furin during MI and the relationship between these changes and prognosis are still unknown. Third, this study did not evaluate the differences between patients with and without MI, and this study could not determine whether MI was associated with higher or lower plasma furin levels. Fourth, the plasma furin activity could not be measured, which may be different from the plasma furin concentration. The degradation rate of the furin substrate is a potential way to detect this; however, the method for detecting plasma furin activity is not established and commercial kits were not available until recently [36]. Lastly, the sample size did not provide enough power to detect differences in endpoints other than the composite MACE outcome.

## Conclusion

The findings of this study suggest that plasma furin is not associated with the risk of MACE, but higher levels of plasma furin may be associated with a higher risk of recurrent MI in AMI patients.

## Supplementary Information

**Additional file 1**: **Table S1**. Spearman correlation analysis between log furin and covariates.

**Additional file 2**: **Figure S1**. Distribution of plasma furin in the sample.

## Data Availability

The datasets that support the findings of this study are available from the corresponding author on reasonable request.

## Abbreviations

AMI: Acute myocardial infarction
AST: Aspartate aminotransferase
ALT: Alanine transaminase
ANP: Atrial Natriuretic Peptide
BMI: Body mass index
cTNT: Troponin T
CK-MB: Creatine kinase-MB
CKD: Chronic Kidney Disease
CV: Cardiovascular
DM: Diabetes mellitus
eGFR: Estimated glomerular filtration rate
ENaC: Epithelial Sodium Channels
HBA1C: Glycosylated hemoglobin A1C
HDL-C: High density lipoprotein cholesterol
LDL-C: Low density lipoprotein cholesterol
LVEF: Left ventricular ejection fraction
MACE: Major adverse cardiac events
NSTEMI: Non-ST-segment elevation myocardial infarction
NT-proBNP: N terminal pro B type natriuretic peptide
PCSK: Proprotein convertase subtilisin/kexin
STEMI: ST-segment elevation myocardial infarction
