# Supplementary material for: The association between plasma furin and cardiovascular events after acute myocardial infarction
Source: BMC Cardiovasc Disord. 2021 Sep 27;21:468. doi: 10.1186/s12872-021-02029-y (PMC8477572; doi:10.1186/s12872-021-02029-y)
Supplement: Supplementary file 1 — Additional file 1: Table S1. Spearman correlation analysis between log furin and covariates. [file 12872_2021_2029_MOESM1_ESM.docx]

**sTable 1. Spearman correlation analysis between log furin and covariates.**

|  | **Age** | **Glucose** | **HbA1c** | **Log eGFR** | **LVEF** | **Log CKMB** | **Log cTNT** | **Log NT-proBNP** |
| --- | --- | --- | --- | --- | --- | --- | --- | --- |
| **Log furin** | 0.007 | 0.022 | -0.007 | 0.035 | -0.003 | 0.051 | 0.065 | 0.018 |
