# Supplementary figures and images for: The association between plasma furin and cardiovascular events after acute myocardial infarction
Source: BMC Cardiovasc Disord. 2021 Sep 27;21:468. doi: 10.1186/s12872-021-02029-y (PMC8477572; doi:10.1186/s12872-021-02029-y)

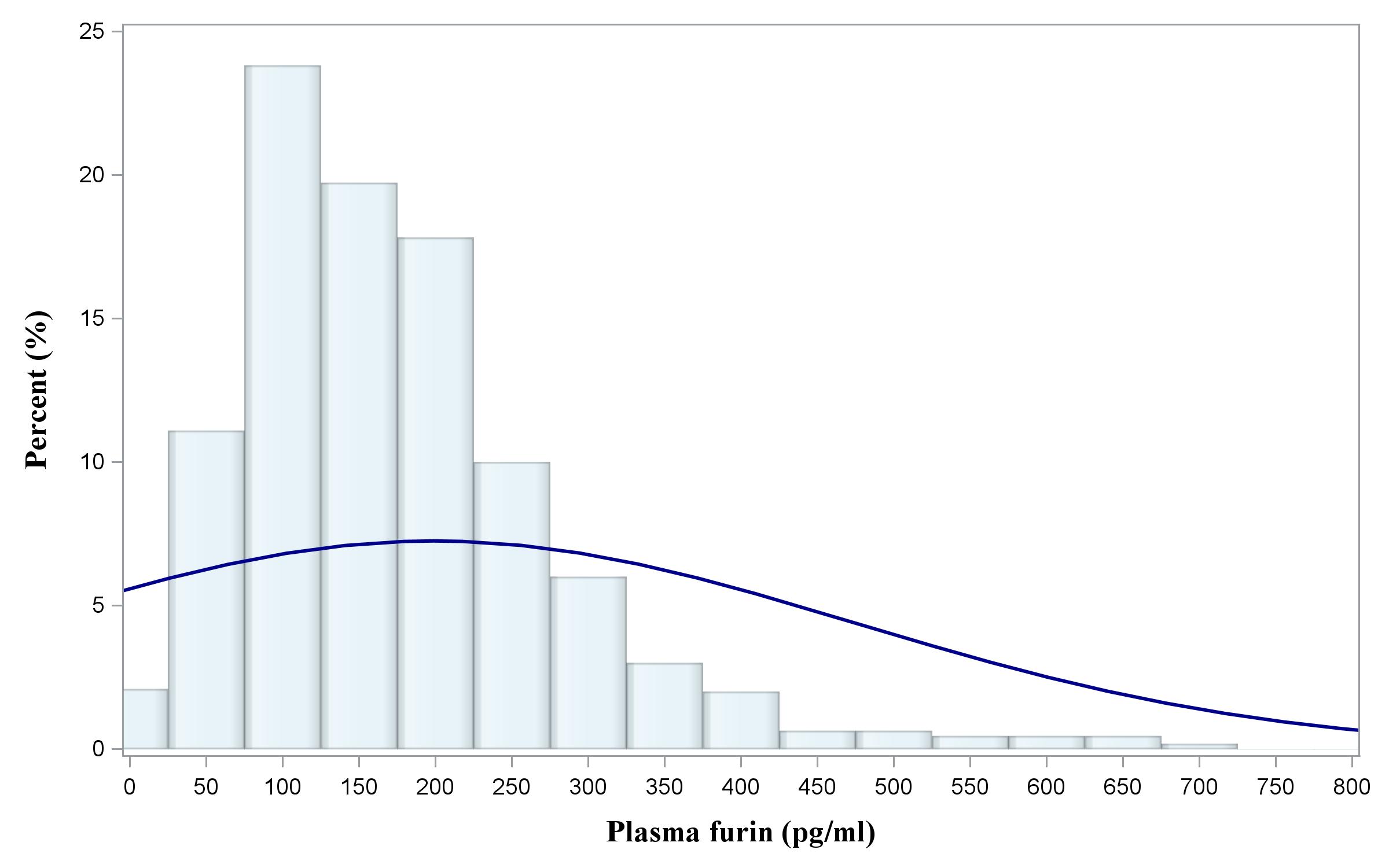

Supplement: Supplementary file 2 — Additional file 2: Figure S1. Distribution of plasma furin in the sample. [file 12872_2021_2029_MOESM2_ESM.jpg]
